# Exploring the use of assistive products to promote functional independence in self-care activities in the bathroom

**DOI:** 10.1371/journal.pone.0215002

**Published:** 2019-04-08

**Authors:** Iván De-Rosende-Celeiro, Gabriel Torres, Mercedes Seoane-Bouzas, Adriana Ávila

**Affiliations:** 1 Department of Health Sciences, University of A Coruña, A Coruña, Spain; 2 Department of Physical and Sports Education, University of A Coruña, A Coruña, Spain; 3 University Hospital Complex of Santiago de Compostela, Santiago de Compostela, Spain; University of Wyoming College of Health Sciences, UNITED STATES

## Abstract

In homes, problems in daily functioning of older people often occur in the bathroom, especially in the transfers to the toilet and/or shower/bath. Assistive products have the potential to maximise functional independence (i.e. performance without assistance from another person) in everyday activities; however, more research is needed to better understand the impact of this technology on independence in the transfers in the bathroom. Additionally, little is known about the role of the environmental factors in the process of implementing bathroom adaptations. Therefore, this cross-sectional study aimed to examine the relationship between the use of assistive products and independence in the transfers in the bathroom. The secondary objective was to determine the role of the environmental factors in predicting the implementation of bathroom adaptations. 193 community-dwelling older adults with disabilities in the basic activities of daily life, who requested public long-term care services in Spain, were included. Data was collected in the participant´s homes using a standardised assessment procedure. There was no significant association between the number of categories of assistive products used in the toilet transfer and the independent performance of this task. In a multivariate model, the number of categories of assistive products used in the transfer to shower/bath was positively associated with the independent performance of this transfer (OR = 2.59, 95%CI = 1.48–4.53; *p* = 0.001). A multivariate analysis revealed that social functioning was significantly associated with the implementation of a bathroom adaptation; social risk was lower in participants who made an adaptation (OR = 0.76, 95%CI = 0.63–0.93; *p* = 0.006). Assistive products may play an important role in promoting independence in the bathroom. Assistive product needs should be addressed when planning community-based interventions aimed at improving daily life. Moreover, social functioning had a strong influence on the installation of bathroom adaptations, suggesting the importance of paying special attention to social factors in the home adaptations planning process.

## Introduction

Disabilities in the basic activities of daily life (ADL) are defined by the need for personal assistance to perform the self-care tasks, i.e. dependence on help from another person in activities such as feeding, dressing, taking a bath or toilet using. In homes, problems in daily functioning of older people often occur in the bathroom, especially in the activity of bathing [1–3]. Thus, a large-scale study concluded that bathing was the first ADL with which older people had difficulty; toileting was in third position [4]. In a longitudinal study, about 60% of community-dwelling older people experienced at least one episode of bathing disability over a six-year period [5]. Disabilities in the self-care activities performed in the bathroom have been associated with future health and social problems. Bathing disability increases the total hours of paid and unpaid personal assistance in homes [6] and the risk of a long-term nursing home admission among older people [7]. Difficulty with bathing is an independent predictor of mortality among community-living older adults [8]; similarly, dependence in toileting is a risk factor for mortality in frail older people [9]. Moreover, people with disabilities and health practitioners agree that maintaining autonomy in toileting activity is essential in order to avoid feelings of loss of self-esteem [10].

Bathing and toileting involve multiple steps or tasks. The literature has divided the activity of bathing into eight categories of tasks [3] and the act of toileting into six tasks [10]. The transfer action is one of the necessary steps to carry out both activities. The research reported in this paper focused on the two transfers in the bathroom area: toilet transfer (getting on and off the toilet) and transfer to the shower/bath. Both transfers are described in the modified version of the Barthel Index [11]. We studied these activities because the transfers are the most problematic tasks in the bathroom [1–3]. More than half of the falls in the bathroom are related to the transfers in community-living older adults [1]. In addition, in the activity of bathing, two studies have shown that the transfer is the task with the highest prevalence rate for dependence [3, 12].

A common strategy for promoting functional independence (i.e. the performance without assistance from another person) in everyday life is to recommend the implementation of adaptations in the physical environment of the bathroom area. In the current study, a bathroom adaptation is an intervention specifically aimed at optimising the functioning of a person in particular and encompasses the installation and the use of equipment and products such as grab bars, raised toilet seats or shower/bath transfer benches. The ISO9999 [13], an internationally recognised classification in the field of disability study, includes the wide range of devices and adaptive equipment to maintain or enhance functional independence in homes within the umbrella term "assistive products". In the definition of the World Health Organization (WHO) [14], this concept refers to “any product, instrument, equipment or technology adapted or specially designed for improving the functioning of a disabled person”. Several previous conceptual models have highlighted the importance of assistive products. The biopsychosocial model of the WHO [14] has highlighted that functioning is determined by the interaction between an individual with a health condition and the context of life, recognising that technology can act as a key facilitator in the person/environment interaction, improving functioning and reducing disability. The Lawton´s Ecological Model [15] established that functional disabilities arise from a mismatch between personal ability and environmental press, noting that assistive products can enhance the person/environment fit. Similarly, the Disablement Process Model [16] postulated that technology can decrease problems in daily functioning by reducing environmental demands on task performance.

Previous theoretical models and clinical convention suggest that assistive products have the potential to improve or maintain functional independence. However, evidence of the effectiveness of assistive products on independence in self-care activities is limited by the small number of studies among community-living older adults. Furthermore, the available findings have shown conflicting results. One randomised controlled trial with frail older people found that an occupational therapy (OT) intervention composed of the provision of assistive products reduced functional decline in ADL [17]. In a prospective study, an OT program based on adaptive equipment improved independence in ADL in a sample of older people [18]. In contrast, this type of intervention did not improve the level of independence in the self-care activities in a clinical trial conducted with individuals requesting home adaptations [19]. Moreover, in the previous research, information on the specific effects of assistive products on the self-care activities performed in the bathroom in older people is very limited. In a longitudinal research, assistive products decreased dependence in bathing but this improvement was not identified in the toileting activity [20]. Lastly, a prospective cohort study with nondisabled older people found that the presence of grab bars or bath seats did not prevent the subsequent development of bathing disability [21].

Despite the potential benefits of technology in daily life, the literature has found high rates of non-use of assistive products [22, 23]. In order to assist in the design of evidence-based strategies aimed at promoting the use of this equipment, it is crucial that professionals know what factors are associated with the utilisation of assistive products. The Matching Person and Technology (MPT) Model [24], one of the most well-known theoretical models in the field of assistive technology, has emphasised the influence of the environmental and personal factors on the use of this type of equipment and devices. Regarding the research on the determinants of the use of assistive products for the self-care activities, previous literature reviews [22, 23, 25] have identified key personal factors such as age, sex or various health conditions; to date, the environmental factors have not been thoroughly examined. In the WHO disability model [14], the environmental factors refer to all conditions of the “external or extrinsic world that form the context of an individual’s life”, including the physical world (e.g. the human-made environments) and the social environment (e.g. social networks and people who provide support). The literature on the impact of the environment has mainly focused on the negative effect of the lack of fit between the assistive product and the characteristics of the physical home environment [22, 23, 25]. However, relatively little is known about the influence of other environmental factors such as social functioning, a broad concept that encompasses the domains of family conditions, social contacts and assistance from the social network [26, 27].

Based on the abovementioned findings, the specific outcomes of assistive products on independence in the transfer tasks in the bathroom among the community-living older population are not known. Additionally, we addressed the factors that contribute independently to the implementation of a bathroom adaptation. Specifically, this research analysed the influence of the factors related to the client´s environment, given that they have been the factors less frequently included in the previous literature. Accordingly, the main objective of this study was to examine the relationship between the use of assistive products and functional independence in the transfers in the bathroom area. The secondary objective was to determine the role of the environmental factors in predicting the implementation of a bathroom adaptation. The research tested two hypotheses: (1) assistive products are positively associated with the independent performance of the transfer tasks, and (2) the environmental factors would account for a portion of the implementation of a bathroom adaptation.

## Methods

### Study design

This descriptive study utilised a cross-sectional design.

### Setting

The study was conducted on a sample of community-living older adults requesting public long-term care services at the regional government office of Coruña, in north-western Spain. This regional office covers the geographically defined area of Coruña, comprising the city of Coruña and six semi-urban and rural municipalities. The study area had, in 2017, a total population of 336 469 inhabitants.

The Spanish long-term care system provides public institutional and community-based services to all those who are legally certified as requiring these type of services. This procedure begins when a person contacts the regional office to request long-term care services. After such a request, the legal certification of long-term care need is determined based on the results of a comprehensive assessment by a trained regional government official (a health professional) in the applicant´s home in accordance with nationally uniform criteria. This highly formalised assessment is known as the *assessment of the dependence status*.

### Participants

The inclusion criteria were as follows: (a) individuals aged 60 years and above; (b) living at home (i.e. not in a residential or nursing care home); (c) requesting public long-term care services at the regional government office of Coruña; and (d) a disability in ADL, which refers to dependence on personal assistance to perform at least one of the following self-care activities of the Barthel Index [28]: eating, bed-chair transfer, toileting, dressing and bathing/showering. People who had a bed-bath or a sponge-bath (in the sink) were excluded.

The data used for this study were originally collected as part of the *assessment of the dependence status*. The administrative records of these official assessments were the source of data for this research. In our study, we performed a detailed retrospective chart review of all the *assessments of the dependence status* completed consecutively by an official of the aforementioned regional government office for 16 consecutive weeks; this official was a trained occupational therapist with extensive experience of working with older people with long-term care needs. During the study period, this official completed 323 *assessments of dependence status* in the study area. Out of all of the 323 assessments reviewed in this study, 193 met the inclusion criteria. These 193 individuals served as the participants in this study. The data set used for this study was provided by the regional government. All data was fully anonymised and kept confidential. Confidentiality was preserved in accordance with the Spanish Data Protection Law. Informed consent was waived because of the retrospective nature of the study. Prior to commencing this research, written ethical approval for this study was granted by the Research Ethics Committee of Galicia.

### Variables

The *assessment of the dependence status* focuses on physical and mental status, ADL performance, social functioning, the use of assistive products and the implementation of home adaptations. Moreover, the primary physician provides written information about the diagnosed health conditions and impairments of the applicant. If the person has cognitive impairment or mental illness, the participation of the primary caregiver in the assessment process is mandatory. The information collected in this official assessment is entered into a standardised electronic record.

In our study, using a data collection sheet, information was recorded on five domains: functional independence in the performance of the two transfers analysed, use of assistive products, implementation of a bathroom adaptation, environmental factors and personal variables. Functional independence in the transfers was the outcome variable for the first objective. Regarding the second objective, the implementation of adaptations in the bathroom area was the outcome variable.

Additionally, to describe the overall level of dependence of the participants, we used the original version of the Barthel Index [28]. It is one of the most widely used rating scales for the measurement of functional independence. This instrument was developed as a measure to assess the degree of personal assistance required to complete 10 ADL. The total score ranges from 0 (total dependence) to 100 (complete independence); a score of 0–20 indicates total dependence, 21–60 severe dependence, 61–90 moderate dependence and 91–99 slight dependence [29].

#### Functional independence in the transfers

A modified version of the Barthel Index [11] was used to assess the performance of the two transfers analysed in this study. Each subject was assessed on his/her activity performance through direct observations. In the current study, the term “functional independence” meant that the individual was completely independent (i.e. without supervision, direction or assistance from another person) in performing the transfer. Dependence meant that another person was involved in the task with personal assistance or directive assistance. In each transfer task, this variable was dichotomised as independent performance vs. personal assistance (dependence).

#### Assistive products

The use of assistive products during the performance of the two transfers studied was assessed. Since this study addressed the assistive products most commonly used in the daily life of the older population, architectural modifications and high technology devices were not included.

Assistive products were classified into five broad categories according to the ISO9999 [13]. Regarding the toilet transfer, two categories were included: assistive products to provide support getting on or off the toilet and products to increase the height of the sitting position. In the transfer to shower/bath, we included three categories: shower stall/unit, grab bar and products for transfer in the sitting position. Based on direct observation, the number of product categories used by the participant in each transfer was determined, ranging from zero to two in the toilet transfer and zero to three in the shower/bath transfer.

#### Bathroom adaptation

Information on the adaptations implemented in the bathroom area was collected. With respect to each of the categories of assistive products used, the participants were asked if the product had been installed specifically for them in the last two years. The primary caregiver answered this question when the participant had cognitive impairment or mental illness. For the current study, an adaptation of the bathroom was defined as the installation of any of the five categories of assistive products analysed in this research in the last two years (at least one category), implemented specifically for the participant; this variable was dichotomous (bathroom adaptation vs. no).

#### Environmental factors

Several factors of the physical and social environment were analysed. The physical environment factors included were the type of living area (densely populated area vs. no) and the home characteristics. Two characteristics of the participant´s homes were evaluated: building year and residential tenure (own vs. rental). We considered three groups of social environment factors: primary caregiver, number of children and social functioning. Regarding the primary caregiver, we included age and the relationship to the participant (offspring vs. other).

Social functioning was assessed using the Gijon´s social-familial evaluation scale (Barcelona-version) [26, 27]. This instrument evaluates three areas of social function: family conditions, social contacts and assistance from the social network. It is simple, easy to administer and allows rapid detection of situations of social risk. The total score ranges from three to 15. High scores identify social risk factors: inappropriate family situation (lack of care, distant family or no family); lack of visits and social contacts; as well as there being no assistance from the social network when necessary. Three categories of social risk have been established: low risk (≤ 7 points), intermediate (8–9 points) and high (≥ 10). The total score correlated positively with the request for a definitive institutionalisation in a nursing home; similarly, the highest scores were an independent predictor of discharge to an institution [26]. Furthermore, the lowest scores on this scale were predictive of home discharge from a geriatric convalescence unit in a prospective cohort study [27].

#### Personal factors

Information on personal factors was collected from the administrative records to identify potential confounding factors. We considered 20 variables, classified into four groups: socio-demographic characteristics (age and gender), body functions (cognition, sensory function and lower limb mobility), the presence or absence of 12 types of diagnosed health conditions and the health care received by the participant. Cognitive functioning was analysed using the Red Cross Mental Scale [30]; according to the previous literature [30], scores ≥ 3 points indicated cognitive impairment. The PULSES-Profile [11] was used to assess sensory function and lower limb mobility. We collected information about the health care received by the participant in the last two years, including rehabilitation interventions (occupational therapy or physical therapy), hospital stay and oxygen therapy.

### Statistical methods

Descriptive statistics were used to summarise the findings. The Kolmogorov-Smirnov test was used to determine the normal distribution. Since the data were not normally distributed, sample characteristics were given either as median and quartiles (Q1-Q3) or as frequency and percentage, as appropriate.

The IBM SPSS 22.0 (Armonk, NY, USA) was used for the statistical analysis. Throughout the study, the level of statistical significance was set a priori at a *p* value of < 0.05 and all tests were two-sided. The statistical methods used to achieve the two study objectives are described below.

#### a. Objective 1: Relationship between assistive products and functional independence in the transfers

The influence of the use of assistive products on independence in the performance of the transfers was assessed. Regarding each of the two transfers studied, the first step was to explore the relationship between the number of product categories used in performing the transfer and functional independence through the non-parametric Mann-Whitney *U*-test; for each transfer, if this association was significant, we chose a multivariate logistic regression analysis with functional independence in the task as binomial outcome variable (independent performance vs. personal assistance) to examine the contribution of assistive products to independence, after adjusting for personal factors as covariates. To avoid overfitting the model, the personal factors included in the multivariate model were those that had statistically significant univariate relationships with the outcome variable. The bivariate associations between functional independence in each transfer and the covariates were tested using the Mann-Whitney *U*-test for the continuous variables and the chi-square test or Fisher´s exact test for the categorical variables.

The results of the multivariate model were expressed as odds ratios (OR) with corresponding 95% confidence intervals (CI); an OR greater than 1.00 indicates a greater probability of the outcome variable and an OR less than 1.00 indicates a lesser probability [31]. A Hosmer-Lemeshow test of the goodness of fit was performed and the Nagelkerke *R*-Square value was calculated.

#### b. Objective 2: influence of environmental factors on the implementation of a bathroom adaptation

Bivariate analyses were carried out to identify the environmental factors associated with the implementation of a bathroom adaptation in the last two years; the relationships were tested by the Mann-Whitney *U*-test, chi-square test or Fisher´s exact test, as appropriate. All statistically significant variables were subsequently entered into a multivariate logistic regression analysis with the implementation of a bathroom adaptation as dichotomous outcome variable (bathroom adaptation vs. no) to explore the contribution of these environmental factors to the bathroom adaptation implemented, after adjusting for all personal factors (covariates) that had statistically significant univariate associations with the outcome variable. The bivariate associations between the implementation of a bathroom adaptation and the covariates were tested through the Mann-Whitney *U*-test, chi-square test or Fisher´s exact test, as appropriate. We presented the results of the multivariate model as OR and 95% CI. The fit of the model was analysed using the Hosmer-Lemeshow goodness of fit test and the Nagelkerke *R*-Square value was calculated.

## Results

### Characteristics of the participants

The median age of the 193 participants was 84 years (Q1-Q3 = 80–89). More than two thirds were women (67.9%). In the Barthel Index, the median score was 65 points (Q1-Q3 = 42.5–80), representing a moderate level of dependence in ADL. On the PULSES-Profile, 85.5% of the participants were dependent in the lower limb mobility activities. More than 26% of the participants had cognitive impairment according to the Red Cross Mental Scale (26.4%). The most common diagnosed health condition was osteoarthritis in lower limb (52.8%). More than 47% of the participants were hospitalised in the last two years (47.7%). In the social-familial evaluation scale, the median score was 8 points (Q1-Q3 = 7–9), representing an intermediate level of social risk.

Data on the use of assistive products for transfers in the bathroom are presented in Table 1. Most participants used at least one assistive product in the performance of the shower/bath transfer (81.9%); this percentage was 12.4% in the toilet transfer.

**Table 1:** Use of assistive products for the transfers in the bathroom area (*n* = 193).

Categories of assistive products	No. participants using the category (%) ^a^
*For transfer to toilet*	
Products to provide support getting on or off the toilet: toilet arm support and/or grab bar/rail	16 (8.3)
Products to increase the height of the sitting position: raised or height adjustable toilet, toilet seat, raised toilet seat mounted on frame and/or toilet seat insert	13 (6.7)
No. of product categories used: median (Q1-Q3)	0 (0–0)
*For transfer to shower/bath*	
Shower stall/unit	123 (63.7)
Grab bar/rail	46 (23.8)
Products for transfer in the sitting position: bath board, bath seat and/or shower/bath transfer bench	31 (16.1)
No. of product categories used: median (Q1-Q3)	1 (1–1)

^a^ Data are presented as *n* (%), unless otherwise stated.

### Outcome data

*a*. *Objective 1*. Regarding the functional independence in the transfers studied, 47.2% of the participants performed the transfer to the toilet independently; in the shower/bath transfer, this data was 37.8%.

*b*. *Objective 2*. A total of 110 participants (57%) implemented a bathroom adaptation in the last two years.

### Main results

#### Objective 1: Relationship between assistive products and functional independence in the transfers

a. *Toilet transfer*. There was no significant association between the number of categories of assistive products used by the participant in the toilet transfer and the independent performance of this task (*p* = 0.317).

b. *Shower/bath transfer*. As shown in Table 2, a significant association was found between greater use of categories of assistive products and the independent performance of the shower/bath transfer (*p* = 0.004).

**Table 2:** Bivariate relationship between independence functional in the performance of the transfer to the shower/bath and personal factors and assistive products (*n* = 193).

	Independent performance (*n* = 73)	Personal assistance (*n* = 120)	*p* value
Personal factors			
*Socio-demographics*			
Age, in years ^a^	83 (80–86)	85 (79–90)	0.054
Gender			
Women	53 (72.6)	78 (65.0)	0.273
Body functions			
Cognition			
Cognitive impairment ^b^	9 (12.3)	42 (35.0)	**0.001**
Sensory function (communication and vision)			
Dependent on assistance/supervision ^c^	10 (13.7)	43 (35.8)	**0.001**
Lower limb mobility			
Dependent in lower limb mobility activities ^c^	54 (74.0)	111 (92.5)	***p*<0.001**
*Health conditions (diagnosed)*			
Osteoarthritis in lower limb	43 (58.9)	59 (49.2)	0.189
Hip fracture	8 (11.0)	8 (6.7)	0.294
Spine fracture	6 (8.2)	8 (6.7)	0.687
Lower limb amputation	0 (0)	3 (2.5)	0.291
Stroke	7 (9.6)	34 (28.3)	**0.002**
Parkinson´s disease	7 (9.6)	13 (10.8)	0.783
Another degenerative neurological disease	5 (6.8)	7 (5.8)	0.768
Ischaemic heart disease	15 (20.5)	22 (18.3)	0.705
Chronic obstructive pulmonary disease	10 (13.7)	22 (18.3)	0.401
Asthma	3 (4.1)	2 (1.7)	0.368
Malignant tumour or haemopathy	7 (9.6)	19 (15.8)	0.218
Pressure sore	0 (0)	3 (2.5)	0.291
*Health care received by the participant* ^d^			
Rehabilitation intervention	2 (2.7)	14 (11.7)	**0.029**
Hospital stay	29 (39.7)	63 (52.5)	0.085
Domiciliary oxygen therapy	2 (2.7)	8 (6.7)	0.324
Assistive products			
*For the shower/bath transfer*			
No. of product categories used ^a^	1 (1–2)	1 (0–1)	**0.004**

Values expressed as *n* (%), unless otherwise stated. All values that are statistically significant are indicated in bold.

^a^ Median (Q1-Q3).

^b^ ≥ 3 points in the Red Cross Mental Scale.

^c^ ≥ 3 points in the PULSES-Profile.

^d^ In the last two years.

Five personal factors were significantly associated with functional independence in the performance of the shower/bath transfer: cognition status (*p* = 0.001), sensory function (*p* = 0.001), lower limb mobility (*p*<0.001), diagnosis of stroke (*p* = 0.002) and a rehabilitation intervention in the last two years (*p* = 0.029) (Table 2).

In the multivariate model adjusting for these five significant covariates, the number of categories of assistive products used by the participant in the shower/bath transfer remained positively associated with functional independence [adjusted OR = 2.59 (95% CI = 1.48 to 4.53); *p* = 0.001; Table 3]. Cognitive impairment (*p* = 0.002) and limitations in the mobility of the lower extremities (*p* = 0.007) were negatively associated with the independent performance of the transfer (Table 3). The model had a good explanatory power, as assessed by the Hosmer-Lemeshow Goodness-of-fit test (*χ*^2^ = 4.99, df = 8, *p* = 0.76). This multivariate model, as a whole, explained 30% of the variance in functional independence in the shower/bath transfer (Nagelkerke *R*-squared = 0.301).

**Table 3:** Multivariate logistic regression predicting independence functional in the performance of the transfer to the shower/bath (*n* = 193).

Independent variables	Odds ratio	95% CI	*p* value
*Body functions*			
Cognitive impairment			
Yes ^a^	0.24	0.10–0.58	**0.002**
No	1.00		
Sensory function (communication/vision)			
Dependent on assistance/supervision ^b^	0.54	0.22–1.30	0.167
Independent	1.00		
Lower limb mobility			
Dependent in lower limb mobility activities ^b^	0.27	0.11–0.70	**0.007**
Independent	1.00		
*Health conditions (diagnosed)*			
Stroke			
Yes	0.50	0.18–1.35	0.169
No	1.00		
*Health care received by the participant* ^c^			
Rehabilitation intervention			
Yes	0.21	0.04–1.08	0.061
No	1.00		
*Assistive products for the shower/bath transfer*			
No. of product categories used	2.59	1.48–4.53	**0.001**

Dependent variable: independent performance = 1; personal assistance = 0 (according to the modified Barthel Index). All values that are statistically significant are indicated in bold.

^a^ ≥ 3 points in the Red Cross Mental Scale.

^b^ ≥ 3 points in the PULSES-Profile.

^c^ In the last two years.

#### Objective 2: influence of environmental factors on the implementation of a bathroom adaptation

Regarding the environmental factors, the social functioning of the participant was significantly associated with the implementation of a bathroom adaptation in the last two years (*p* = 0.010; Table 4). The bivariate analyses found that four personal factors were significantly associated with a bathroom adaptation (Table 4): osteoarthritis in lower limb (*p* = 0.046), Parkinson’s disease (*p* = 0.028), rehabilitation intervention (*p* = 0.010) and hospital stay (*p* = 0.028).

**Table 4:** Bivariate relationship between the implementation of a bathroom adaptation (in the last two years) and personal and environmental factors (*n* = 193).

	Bathroom adaptation (*n* = 110)	No (*n* = 83)	*p* value
Personal factors			
Age, in years ^a^	84 (79.75–89)	85 (81–89)	0.490
Women	71 (64.5)	60 (72.3)	0.254
Cognitive impairment ^b^	30 (27.3)	21 (25.3)	0.758
Sensory function: dependent on assistance/supervision ^c^	26 (23.6)	27 (32.5)	0.171
Dependent in lower limb mobility activities ^c^	97 (88.2)	68 (81.9)	0.222
Osteoarthritis in lower limb	65 (59.1)	37 (44.6)	**0.046**
Hip fracture	11 (10.0)	5 (6.0)	0.321
Spine fracture	6 (5.5)	8 (9.6)	0.267
Lower limb amputation	3 (2.7)	0 (0)	0.261
Stroke	23 (20.9)	18 (21.7)	0.896
Parkinson´s disease	16 (14.5)	4 (4.8)	**0.028**
Another degenerative neurological disease	8 (7.3)	4 (4.8)	0.485
Ischaemic heart disease	14 (12.7)	7 (8.4)	0.343
Chronic obstructive pulmonary disease	16 (14.5)	16 (19.3)	0.382
Asthma	3 (2.7)	2 (2.4)	0.891
Malignant tumour or haemopathy	17 (15.5)	9 (10.8)	0.353
Pressure sure	3 (2.7)	0 (0)	0.261
Rehabilitation intervention	14 (12.7)	2 (2.4)	**0.010**
Hospital stay	60 (54.5)	32 (38.6)	**0.028**
Domiciliary oxygen therapy	5 (4.5)	5 (6)	0.748
Environmental factors			
*Primary caregiver*			
Relationship to the participant			
Offspring ^d^	65 (59.1)	51 (61.4)	0.741
Age			
Under 65 years old	75 (68.2)	62 (74.7)	0.323
*No*. *of children* ^a^	2 (1–2)	2 (1–2)	0.487
*Type of living area*			
Densely populated area ^e^			
Yes	54 (49.1)	46 (55.4)	0.384
*Home*			
Building year			
Before 1990	99 (90.0)	71 (85.5)	0.344
Residential tenure			
Own ^f^	100 (90.9)	72 (86.7)	0.358
*Social functioning*			
Gijon´s social-familial evaluation scale ^a^	7 (7–9)	8 (7–10)	**0.010**

Values expressed as *n* (%), unless otherwise stated. All values that are statistically significant are indicated in bold.

^a^ Median (Q1-Q3).

^b^ ≥ 3 points in the Red Cross Mental Scale.

^c^ ≥ 3 points in the PULSES-Profile.

^d^ Offspring vs. spouse, sibling, other kin, friend, other.

^e^ According to the Galician Institute of Statistics: density > 500 inhabitants/km^2^ and a population size > 50,000 inhabitants.

^f^ Own vs. rental.

In the multivariate model adjusting for these four significant covariates, we observed an inverse relationship between the scores on the Gijon´s social-familial evaluation scale and the implementation of a bathroom adaptation; social risk was significantly lower in participants who made an adaptation of the bathroom [adjusted OR = 0.76 (95% CI = 0.63 to 0.93); *p* = 0.006; Table 5]. Osteoarthritis in lower limb (*p* = 0.008), Parkinson’s disease (*p* = 0.012) and rehabilitation intervention (*p* = 0.042) were positively associated with the outcome variable (Table 5). A Hosmer-Lemeshow test of the goodness of fit produced a chi-square value of 4.73 (df = 8, *p* = 0.79), suggesting that the model had a good explanatory power. The multivariate model explained 19% of the variance in the implementation of a bathroom adaptation (Nagelkerke *R*-squared = 0.196).

**Table 5:** Multivariate logistic regression predicting the implementation of a bathroom adaptation (*n* = 193).

Independent variables	Odds ratio	95% CI	*p* value
*Health conditions* (diagnosed)			
Osteoarthritis in lower limb			
Yes	2.36	1.25–4.45	**0.008**
No	1.00		
Parkinson´s disease			
Yes	4.66	1.41–15.38	**0.012**
No	1.00		
*Health care received by the participant*			
Rehabilitation intervention			
Yes	5.30	1.07–26.37	**0.042**
No	1.00		
Hospital stay			
Yes	1.72	0.90–3.27	0.100
No	1.00		
*Social functioning*			
Gijon´s social-familial evaluation scale ^a^	0.76	0.63–0.93	**0.006**

Dependent variable: implementation of a bathroom adaptation (in the last two years) = 1; no = 0. All values that are statistically significant are indicated in bold.

^a^ Higher scores reflect greater social risk.

## Discussion

The core contributions of this study to current knowledge about the use of assistive products in the homes of older people were two. First, in support of our hypothesis, the findings suggested that assistive products have a positive impact on functional independence in the bathroom. This type of technology was associated with the independent performance of the shower/bath transfer, which is one of the most demanding self-care tasks in the daily life of this population group. Secondly, we provided detailed information about the role of environmental factors, not normally gathered in research in the field of assistive products. To our knowledge, this was the first research that showed that social functioning has an important influence on the bathroom adaptation process.

The use of this type of equipment was a strong predictor of independence in the shower/bath transfer. People who used more categories of assistive products were less likely to receive personal help. This result could be explained by the facilitating effect of this technology in everyday life. Assistive products have the potential to facilitate transfers and to compensate for functional disabilities due to impairments such as problems in balance, reduced muscle power, coordination problems or diminished respiratory function. In the bathroom, this equipment can reduce pain and the level of difficulty; moreover, assistive products promote energy conservation, comfort and safety, helping to prevent falls [19, 32–35]. Accordingly, these technologies may enable the individual to perform tasks that would otherwise be difficult or impossible. The results showed that assistive products seem to play an important role in reducing personal assistance in the bathroom area, which allows participants to live more independently in their own homes and, consequently, individuals may remain at home for a longer period of time. In addition, it should be noted that the assistive products analysed in our study are low-technology devices and are characterised by ease of use, so the use of this equipment in daily life does not usually require specialised training or the help of an informal caregiver [32]. Therefore, the study appears to support the inclusion of assistive products in the theoretical models explaining functional independence [14–16]. Finally, our findings are in line with the improvement in the level of independence in the bathing activity found in a research on the effects of assistive products [20]; however, although most of the devices were related to the shower/bath transfer, the specific effect of assistive products on the transfer task was unknown due to the use of a global measure of independence for the whole of the bathing activity. Moreover, comparison of results between studies is difficult due to the heterogeneity of the samples studied, of the product categories included in each research, and of the instruments used to assess independence.

Assistive products were not statistically associated with the independent performance of the toilet transfer. Several reasons might explain this finding. First, a high proportion of participants performed this task independently, consistent with previous studies that have found higher rates of independence in toileting activity than in bathing [1, 4, 20]; thus, the technology for the toilet transfer may be more effective in samples with higher functional dependence levels. Furthermore, only a few participants used assistive products in this transfer, in line with the high rates of underutilisation of equipment for the toilet transfer found in the literature [17, 19, 36]. Another possible explanation is that the dichotomisation of the dependent variable (independence vs. personal assistance) did not allow discrimination between the different levels of personal help. It may be that the effect of assistive products consists in reducing the degree of assistance received (e.g. an improvement from total assistance to minimal physical help), without necessarily achieving complete independence in the transfer. Additionally, further research should examine whether assistive technology is more effective on other dimensions of daily functioning beside functional independence, such as the degree of difficulty in performing an activity. In a large sample of older people, considerable proportions of subjects were independent in most ADL, while high proportions reported difficulties in completing these activities [37]. In line with our study, the use of assistive products did not improve functional independence in ADL in a randomised clinical trial involving community-dwelling adults [19]; however, these products decreased levels of difficulty in ADL significantly, especially in self-care activities performed in the bathroom such as the toilet transfer [19].

Social functioning contributed in a significant way to explaining the implementation of bathroom adaptations. It was studied by means of a screening instrument, whose application allowed analysing the degree of social risk. We showed that a low social risk was associated with the installation of assistive products in the bathroom area. These findings confirmed the positive influence of three social factors: living with the spouse or other family members without conflict, the existence of good social contacts outside the home and visits of friends and other significant persons from the community context, and receiving sufficient assistance from the social network. Given that these factors are indicative conditions of a social support context [38–42], the multivariate model placed it as a potentially determining variable in the implementation of bathroom adaptations. Defined as “the interactive process through which the individual obtains emotional, instrumental or economic help from the social network” [43], social support is a multidimensional construct. It includes various types of functional support: emotional (the expression of affection), informational (advice and feedback) and instrumental/tangible (physical assistance, e.g. help with transportation, and financial support) [39, 40, 42]. Through these categories of supportive behaviour and social exchange, previous studies have found that social support favours health and well-being [44, 45]. Consistent with our findings, several literature reviews [46, 47] and meta-analyses [45, 48, 49] have shown the relationship between an optimal level of social support and compliance with therapeutic recommendations or the adoption of health promotion behaviours. Finally, the impact of social functioning on bathroom adaptations found in this research corroborated the theoretical principles of the MPT Model [24] described in the introduction section, confirming the importance of paying special attention to social factors in the home adaptations planning process.

Home adaptation is a complex procedure whose implementation involves the execution of multiple actions: detection of individual needs, contact with several specialised professional profiles, visits to orthopaedics or companies in the sector, selection of the right device, search for financing options and the purchase and installation of the selected assistive products [50]. Although the causal mechanisms of the interrelations among social functioning and the bathroom adaptations should be elucidated in further studies, the physical and mental status of participants and the diversity of the actions to be carried out for the implementation of home adaptations could explain this association. The study population was characterised by advanced age, severe limitations in motor skills and a high prevalence of impaired cognitive functions. Given the complexity of the home modification process and the remarkable functional dependence of the participants, it is plausible that the population studied is more likely to need assistance from the social environment to make these adaptations. Significant people in the social context such as caregivers may promote the implementation of a bathroom adaptation through the development of facilitating actions included within the different dimensions of social support; for example, they can assist in tasks such as requesting information from a professional, providing understandable information and guidance, executing some of the necessary steps for the implementation and participating in the financing.

These results extend the existing evidence base for this intervention modality, but should be interpreted carefully because of the following limitations. The reader must keep in mind that the study was cross-sectional, so the findings cannot demonstrate a causal association between variables. However, we believe that the findings give a great deal of information as a basis for further research. Longitudinal studies are needed to establish the temporal validity of any of the associations found. Moreover, the sample size was relatively small, thus reducing the statistical power of the calculations and increasing the risk of not detecting significant associations. Lastly, another limitation was the representativeness of the sample. The participants were a non-probability convenience sample; in addition, the sample consisted solely of older adults who were applicants for public long-term care services in a localised geographical area and most participants were individuals aged > 80 years. The relatively small number of participants, the sample characteristics and the fact that the population was selected using a non-random sampling technique limited the generalisability of the current findings to all Spanish community-dwelling older adults. Consequently, further research to substantiate these preliminary findings is warranted using large-scale and population-based prospective designs.

This study has important clinical implications for practice in community settings. Assistive technology device needs should be addressed when planning community-based health and social programs aimed at improving daily functioning. Environmental strategies to maximise independence in daily life could include a systematic assessment of technology needs, individualised advice about the services of provision of assistive products, training actions on the appropriate use of the devices, as well as grants and leasing systems to facilitate access to assistive products. Furthermore, the literature in the field of assistive technology has highlighted the importance of carrying out periodic assessments of the assistive product needs due to the appearance of changes in the functional abilities of the older population over time. For example, one randomised controlled trial found a loss of functional independence over time in a sample of community-dwelling older adults, although people who used assistive products for everyday activities showed less functional decline than those in the non-user group [17]. Fänge and Iwarsson (2005) [20] showed that 26% of the participants who received technology for home adaptation requested a grant for a new adaptation in a period of nine months, corroborating that the needs of assistive products change in short periods of time. On the other hand, given the potential influence of social functioning on the implementation of bathroom adaptations, consideration of social risk factors should be present in the decision-making process related to the planning of home adaptation programs. Accordingly, the socio-familiar situation, the client´s contacts and the support received from the social network should be investigated in the assessment process of the strategies based on the use of equipment for daily life.

In conclusion, the findings showed that the use of assistive products is a relevant facilitating intervention that has a positive impact on the performance of the shower/bath transfer. This technology may play an important role in promoting functional independence in the bathroom area so that assistive products may be beneficial as a supplementary tool in health and social care for older people with disabilities in ADL living in the community. Moreover, social functioning seems to have a strong influence on the installation of bathroom adaptations, suggesting the importance of paying special attention to social factors in the process of implementing this type of home adaptations.

## Supporting information

S1 Dataset (XLSX)
